# Direct LC-MS/MS Analysis of Extra- and Intracellular Glycerophosphoinositol in Model Cancer Cell Lines

**DOI:** 10.3389/fimmu.2021.646681

**Published:** 2021-03-02

**Authors:** Ana Margarida Campos, Genoveffa Nuzzo, Alessia Varone, Paola Italiani, Diana Boraschi, Daniela Corda, Angelo Fontana

**Affiliations:** ^1^Consiglio Nazionale delle Ricerche, Institute of Biomolecular Chemistry, Pozzuoli, Italy; ^2^Consiglio Nazionale delle Ricerche, Institute of Biochemistry and Cell Biology, Naples, Italy; ^3^Consiglio Nazionale delle Ricerche, Department of Biomedical Sciences Rome, Italy; ^4^Department of Biology, University of Naples Federico II, Naples, Italy

**Keywords:** inflammation, cancer, mass spectrometry, autocrine and paracrine mechanism, solid phase extraction, second messenger, lipid metabolism

## Abstract

Glycerophosphoinositols (GPIs) are water-soluble bioactive phospholipid derivatives of increasing interest as intracellular and paracrine mediators of eukaryotic cell functions. The most representative compound of the family is glycerophosphoinositol (GroPIns), an ubiquitous component of mammalian cells that participates in cell proliferation, cell survival and cell response to stimuli. Levels and activity of this compound vary among cell types and deciphering these functions requires accurate measurements in *in vitro* and *in vivo* models. The conventional approaches for the analysis of GroPIns pose several issues in terms of sensitivity and product resolution, especially when the product is in the extracellular milieu. Here we present an UPLC-MS study for the quantitative analysis of this lipid derivative in cells and, for the first time, culture supernatants. The method is based on a solid-phase extraction that allows for fast desalting and analyte concentration. The robustness of the procedure was tested on the simultaneous measurements of intra- and extracellular levels of GroPIns in a number of human cell lines where it has been shown that the non-transformed cells are characterized by high extracellular level of GroPIns, whereas the tumor cells tended to have higher intracellular levels.

## Introduction

Phospholipids, which constitute the basic structure and organizational milieu of the cell membrane, have gained increasing interest because of their active role in diverse cell functions (1). This is particularly true for the phosphoinositides, a minor fraction of the total membrane phospholipids characterized by specific subcellular distribution and by a rapid and regulated turn-over that is at the basis of their participation in a number of events, including endocytosis, secretion, and autophagy (2). Phosphoinositides are substrates of phospholipases, phosphatases and kinases, which generate several phosphorylated phosphoinositols. These are crucial in the activation of signaling pathways and docking with proteins or protein complexes that regulate cellular processes such as cytoskeleton organization, cell spreading, and intracellular membrane traffic (3–5).

For the past years, studies have focused on glycerophosphoinositols (GPIs), a class of phospholipid metabolites derived from membrane phosphoinositides by two successive deacylation steps catalyzed by phospholipase A_2_IVα (PLA_2_IVα) (6). The cellular levels of GroPIns, the most representative GPI, range from few micromolar to almost millimolar concentrations (7–9), depending on cell type and cell state in terms of cell differentiation, activation or oncogenic transformation (7, 10–14). GroPIns is also present in the extracellular space since it can cross the plasma membrane via the permease Glut2, identified as a GroPIns-specific transporter in mammals (15). Supporting the hypothesis of an autocrine/paracrine role for this metabolite, it was observed that exogenously added GroPIns can elicit thyroid-stimulating hormone (TSH)-independent cell growth of the follicular thyroid cell line PCCl3 (6). Similarly, treatment of A375MM (human melanoma) and MDA-MB-231 (human mammary carcinoma) cells with GroPIns inhibited their migration in *in vitro* models of extracellular matrix invasion (16). Furthermore, GroPIns acted as an anti-inflammatory factor by blocking the signaling cascade triggered by LPS in primary human monocytes, including NF-κB translocation to the nucleus (17).

Intracellular levels of GroPIns were initially measured by radioisotope labeling (6, 11, 15, 18, 19) and, more recently, by mass spectrometry (MS) (8, 9). However, GroPIns is a water-soluble charged metabolite, and its analysis by conventional chromatographic protocols poses several problems in terms of resolution and reproducibility. In addition, the MS analysis of water-soluble small biomolecules is often hampered by matrix effects due to the presence of inorganic salts, mostly sodium phosphate or sodium chloride, that decrease both stability of the ionization process and yield of protonated ions because of extensive sodium ion adduction (20–22).

The aim of the present study is to implement a robust ultra performance liquid chromatography-tandem mass spectrometry (UPLC-MS/MS) method for a quantitative analysis of GroPIns from cell pellets and extracellular fluids. In order to overcome the technical issues that have so far frustrated the direct measurement of this molecule in cell supernatants and extracellular milieu by MS-based techniques, we sought a pretreatment of the sample allowing for both fast desalting and concentration of the analyte. To test the reliability and robustness of the new methodology, the experimental procedure was applied to the analysis of GroPIns in different human cell lines under different conditions, including A375MM cells, a human melanoma cell line already used in functional study of this lipid mediator (9).

## Materials and Methods

### General

The sodiated form of GroPIns was obtained from Echelon Biosciences (Salt Lake City, UT, USA). Ammonium hydroxide solution (≥25% in water, eluent additive for LC-MS) and formic acid solution (98–100% in water, eluent additive for LC-MS), EGF, insulin, cholera toxin, hydrocortisone, calcium chloride dehydrate, potassium chloride, sodium bicarbonate, sodium chloride, sodium phosphate dibasic heptahydrate, sodium phosphate monobasic monohydrate were obtained from Sigma-Aldrich, Inc. (St. Louis, MO, USA). Magnesium chloride hexahydrate was obtained from VWR Chemicals (VWR International Srl, Milano, Italy). HPLC grade acetonitrile and methanol were purchased from Merck (Darmstadt, Germany). Super purity acetic acid was purchased from Romil (Cambridge, UK). Chromabond^®^ HR-XA SPE columns were obtained from Macherey-Nagel GmbH & Co. KG (Düren, Germany). For cell culture media: Dulbecco's Modified Eagle Medium (DMEM), DMEM/Nutrient Mixture F12 (DMEM-F12), Roswell Park Memorial Institute 1640 medium (RPMI-1640), fetal bovine serum and horse serum were all from Gibco (Thermo Fischer Scientific, Waltham, MA, USA); penicillin, streptomycin and L-glutamine were from Sigma-Aldrich, Inc. The cPLA_2_α inhibitor (N-{(2S,4R)-4-(Biphenyl-2-ylmethyl-isobutyl-amino)-1-[2-(2,4-difluorobenzoyl)-benzoyl]-pyrrolidin-2-ylmethyl}-3-[4-(2,4-dioxothiazolidin-5-ylidenemethyl)-phenyl]acrylamide, HCl) was obtained from Calbiochem (San Diego, CA, USA). All other cell culture reagents were of the highest purity and purchased from Gibco.

### Cells and Culture Conditions

The human cell lines used in this study were the prostate adenocarcinoma cell line PC-3 (ATCC^®^ CRL-1435™), the SV40-immortalized prostate epithelial cell line PNT2 obtained from Dr. Alfredo Budillon (Istituto Nazionale Tumori IRCCS – Fondazione Pascale, Napoli, Italy), the breast adenocarcinoma cell line MDA-MB-231 (ATCC^®^ HTB-26™), the near diploid non-tumorigenic breast epithelial cell line MCF-10A (ATCC^®^ CRL-10317), and the metastatic variant of skin melanoma cell line A375MM, obtained from the Institute of Oncological Research in Barcelona through the Egea laboratory at the Barcelona University. Cells were maintained in culture medium (DMEM-F12 for PC-3 and A375MM, RPMI-1640 for PNT2, DMEM for MDA-MB-231) supplemented with 10% fetal bovine serum, 100 U/mL penicillin, 0.1 mg/mL streptomycin and 2 mM L-glutamine. MCF-10A cells were maintained in DMEM-F12 supplemented with 5% horse serum, 20 ng/mL EGF, 500 ng/mL hydrocortisone, 100 ng/mL cholera toxin, 10 μg/mL insulin and 1% L-glutamine.

For GroPIns extraction, cells were cultured in 10 cm Petri dishes for 24 h to reach 70–80% confluency. The extracellular medium (10 mL) was collected by aspiration and frozen at −80° C until analysis. Cells were then washed twice with ice-cold PBS and briefly treated with trypsin-EDTA (Sigma-Aldrich, Inc.). Detached cells were collected by centrifugation and the resulting pellet was stored at −80° C until analysis. For the inhibition assay, A375MM cells were incubated with 0.5 μM cPLA_2_α inhibitor for 16 h and then collected as described above.

### Preparation of the Isotonic Solution for SPE Tests

A medium free of organic components and isotonic (156.50 mM) to DMEM-F12 cell culture medium was prepared with 1.05 mM CaCl_2_, 0.3 mM MgCl_2_, 0.41 mM MgSO_4_, 4.16 mM KCl, 29.02 mM NaHCO_3_, 120.61 mM NaCl, 0.50 mM Na_2_HPO_4_, and 0.45 mM Na_2_HPO_4_-H_2_O in 1 L milli-Q water. Salts were weighted in anhydrous form, added to water and vigorously mixed until complete dissolution at room temperature.

### Solid Phase Extraction Elution

Cell pellets (1.9 × 10^5^ cells for MCF10A; 5.8 × 10^6^ for MDA-MB-231; 2.2 × 10^6^ for A375MM; 8.0 × 10^6^ for PC3; 7.0 × 10^6^ for PNT2) were resuspended in 2 mL milli-Q water, vortexed and sonicated in ice (3 ×30 s with a 10 s break in between). The slurry suspension was loaded onto the CHROMABOND^®^ HR-XA prepacked column previously washed with 6 mL milli-Q water and 6 mL MeOH. Elution started with 6 mL milli-Q water followed by 6 mL MeOH and 2 mL 2% formic acid. Finally, GroPIns was collected by other 6 mL of 2% formic acid and directly analyzed by UPLC-MS/MS (injection volume 2 μL) as described below.

For the analysis of the extracellular medium, 1 mL cell supernatant (1/10 of the collected volume) was diluted with 1 mL milli-Q water and then processed by SPE on CHROMABOND^®^ HR-XA prepacked columns as described above for cell pellets. Salts and other soluble components of the medium were washed off in the fraction eluted by 6 mL milli-Q water, while GroPIns was collected in the last fraction eluted by 6 mL 2% formic acid solution. This material was directly analyzed by UPLC-MS/MS (injection volume 2 μL). The analysis was performed in technical triplicates unless otherwise mentioned.

### UPLC-MS Analysis

Analysis of GroPIns was achieved by a modification on the UPLC tandem MS method described by Grauso et al. (9) in order to adapt the procedure to the Orbitrap technology that allows acquisition of high resolution, mass accurate data. Briefly, the analysis was carried out on a Waters (Milford, MA, USA) ACQUITY UPLC BEH Amide column (100 × 2.1 mm, 1.7 μm) at 30°C, using 0.01% ammonium hydroxide (pH 9)/acetonitrile 95:5 (v/v) (solvent A) and 100% acetonitrile (solvent B) as mobile phases. The elution conditions employed a flow rate of 0.3 mL/min, that is slightly slower than that used in Grauso et al. (9), with 7.5% solvent A for 2 min followed by a first gradient from 7.5% solvent A to 52.5% solvent A over 4.6 min and a second gradient from 52.5 solvent A to 7.5% of solvent A in 3.4 min. The whole run accounted for a total time of 10 min and was followed by inter-run equilibration with 7.5% solvent A for 10 min. MS detection was performed on a Q-Exactive Orbitrap in negative ion mode. MS source parameters were as follows: electrospray voltage 3.20 kV, capillary temperature 320°C, s-lens rf level 45, auxiliary gas flow rate 25, sheath gas flow rate 30. Full MS scans were acquired within 150–400 m/z with a mass resolution of 70,000. The target value (AGC) was 1e^6^ and the maximum allowed accumulation time (IT) was 100 ms.

The analysis was carried out by injecting 2 μl of 2% formic acid fraction collected by SPE of cell pellets and culture supernatants. No pretreatment of the SPE fractions was carried out prior to UPLC-MS/MS analysis. GroPIns eluted at a retention time of 5.99 min.

### Statistical Analysis

Statistical analysis was performed using GraphPad Prism 5 (GraphPad Software, San Diego, CA; www.graphpad.com). Two-way analysis of variance (ANOVA) with the Bonferroni *post-hoc* test was used to determine significant differences among samples.

## Results and Discussion

### Mass Spectrometry and External Calibration

**Figure 1:**
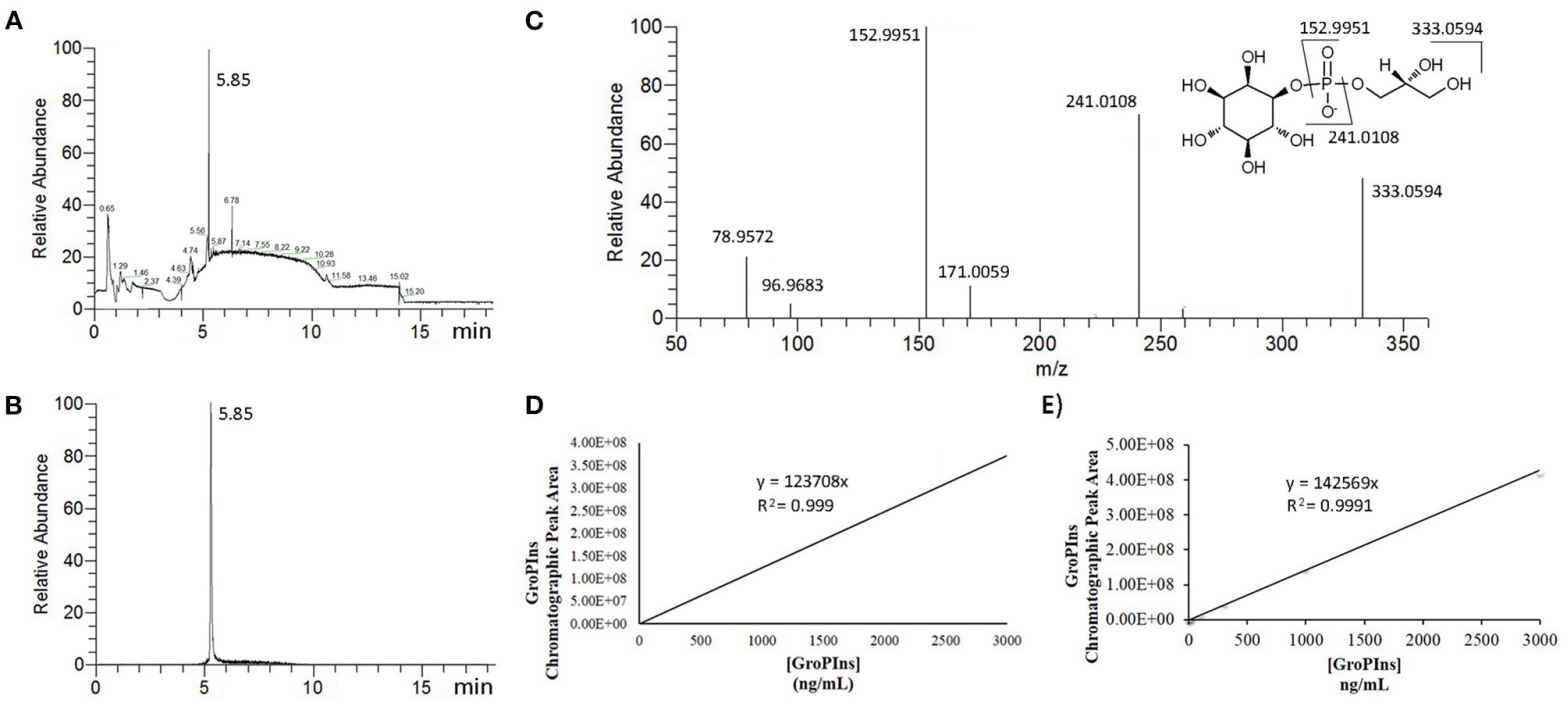
MS Analysis of GroPIns. **(A)** Total ion chromatographic profile of cell extracts (MDA-MB-231); **(B)** Selected ion chromatogram (HRESI^−^ *m/*z 333.0594) of GroPIns; **(C)** MS/MS spectrum of GroPIns: HRESI^−^ *m/z* 333.0593 [M]^−^, 241.0108 [M-glycerol]^−^, 152.9951 [M-inositol]^−^, 96.9683 [H_2_${\text{PO}}_{4}^{-}$]^−^ and 78.9572 [PO_3_]^−^; **(D)** calibration curve in milli-Q water; **(E)** calibration curve in 2% formic acid.

For the UPLC-MS/MS analysis of GroPIns, we adapted our previous method (9) to the Orbitrap technology. Reduction of the flow rate increased the time of the elution steps on a reversed-phase amide column stable from pH 2 to 12 but improved the reproducibility of the process and peak symmetry (Figures 1A,B). Furthermore, in comparison to the previous method on a triple-quadrupole mass spectrometer (9), we achieved a slightly increase of sensitivity together with high resolution (HR) mass data that improved accuracy on the molecular and fragmentation peaks. Accordingly, the MS analysis showed a main molecular ion [M]^−^ at *m/z* 333.0593 (calculated for C_9_H_18_O_11_P^−^, 333.0592) and four MS/MS characteristic fragments at *m/z* 241.0115 [M-glycerol]^−^, *m/z* 152.9947 [M-inositol]^−^, *m/z* 96.9682 [H_2_${\text{PO}}_{4}^{-}$]^−^ and *m/z* 78.9576 [PO_3_]^−^ (Figure 1A) that were used for the unambiguous identification of the analyte (Figure 1C).

The absence of deuterated analogs of GroPIns has always been one of the main obstacles in the accurate quantification of this lipid derivative. To overcome this limitation, external GroPIns calibration curves were obtained using standard solutions of GroPIns at different concentrations both in water (Figure 1D) and 2% formic acid (Figure 1E). As reported in Figure 1, the calibration curves showed an excellent linearity in the range between 3 and 3,000 ng/mL in both solvents (see also, Supporting Material for replicates). The lower limit of quantification for GroPIns was 3 ng/mL (8.98 nM) under both conditions. However, for the analysis (see below), we have always used 2% formic acid conditions to build the calibration curves.

### SPE and UPLC-MS/MS Method

Several strategies have been developed for desalting samples prior to ESI-MS analysis. Most of these methods are suitable for large or lipophilic molecules but generally they cannot be applied to small, hydrophilic molecules. Because of its high solubility, an approach based on polarity or solubility is not capable of concentrating GroPIns in a salt-free fraction. However, GroPIns is an acidic compound (pKa = 1.83) (23) with a negative charge at neutral pH, which can be used to selectively bind GroPIns to an anion exchanger while salts are washed off. Thus, different commercial ion-exchange resins were tested to concentrate GroPIns at neutral pH from a solution of 500 ng/mL of analyte in milli-Q water (data not shown). The best result was achieved by a strong anion exchanger solid phase extraction (SPE) cartridge, namely CHROMABOND^®^ HR-XA, containing a spherical polystyrene/divinylbenzene (PS/DVB) sorbent with quaternary ammonium groups as exchanging centers. In agreement with Brousmiche et al. (24), the column was first eluted with milli-Q water, followed by MeOH and finally 2% formic acid. This last fraction was recovered and directly analyzed by the above-described UPLC-MS/MS method. The analysis showed a single chromatographic peak of GroPIns, with an overall recovery of 94.4 ± 11.6% according to the external calibration curve (Table 1).

**Table 1:** Recovery yields of GroPIns in cell-free media by SPE method.

**Sample**	**Dilution factor**	**Replicates (*n*)**	**Spiked GroPIns (ng/mL)**	**Recovered GroPIns (ng/mL)^d^**	**% Recovery^d^**
Milli-Q water	–	3	500.0	442.7 ± 16.1	94.4 ± 11.6
Culture medium^a^	–	3	500.0	3.2 ± 1.8	0.6 ± 0.4
Isotonic solution^b^	–	3	500.0	9.96 ± 3.7	1.99± 1.2
Diluted isotonic solution^b^^,^^c^	2	3	250.0	210.2 ± 11.2	84.1 ± 5.0
Diluted culture medium^a^^,^^c^	2	8	250.0	236.0 ± 8.4	94.4 ± 3.4

a *DMEM-F12 culture medium supplemented with serum, antibiotics and glutamine*.

b *See the Materials and Methods section for the composition of the isotonic solution*.

c *Diluted 1:2 v/v in milli-Q water*.

d *Values are reported as mean ± SD*.

To test this procedure, 1 mL DMEM-F12 culture medium supplemented with serum, antibiotics and glutamine (complete medium) was spiked with 500 ng GroPIns and submitted to the same SPE elution protocol. Analysis of the last fraction by UPLC-MS/MS showed that only minor amounts (<1% of the spiked amount) of GroPIns were recovered (Table 1). To assess whether the salts or other components of the complete medium interfered with the SPE, we added 500 ng GroPIns to an isotonic solution to DMEM-F12 i.e., a solution containing the same salt composition as DMEM-F12. SPE and UPLC-MS analysis of this solution gave a GroPIns recovery yield lower than 2% (Table 1), thus indicating that salt concentration was the main factor affecting the elution of the lipid mediator. At high concentrations, salt anions compete with GroPIns for the positive charges of the quaternary ammonium groups on the sorbent and reduced the affinity of the resin for the molecule. Since the reduction of the salt loading on the column could solve this technical issue, serial dilutions of the culture medium spiked with GroPIns by milli-Q water were performed from 9:1 to 1:4 (v/v) (recovery has not shown). The best result was obtained by a 1:2 (v/v) dilution (one volume of medium with one volume of milli-Q water) that, after SPE processing and UPLC-MS analysis, yielded 210.2 ± 12.5 ng (*n* = 2) of the total 250 ng of GroPIns loaded onto the column (~84% recovery). To confirm the efficiency and reliability of the procedure, eight samples of 250 ng GroPIns were spiked in 1 mL complete medium and diluted with 1 mL milli-Q water. The final solutions (2 mL each) were processed according to the SPE elution protocol reported above. The last fractions of these extractions were collected and analyzed by UPLC-MS to give an overall recovery of 94.4 ± 3.4% (*n* = 8) of GroPIns (Table 1).

### Detection of Extracellular and Intracellular GroPIns

GroPIns regulates intracellular signaling and is involved in several different cellular processes, including cell growth and differentiation (6, 14, 19). Buccione et al. observed that GroPIns, unlike other inositol-containing molecules, may prevent *in vitro* tumor cell migration (16). More recently, the related metabolite GroPIns4P has been reported to activate fibroblast migration by a cPLA_2_-dependent pathway that might have broad implications in immune-inflammatory response and tumor progression (25). On the basis of these observations, a paracrine function of this family of lipid mediators has been also put forward for cell-to-cell communication in immunity and cancer (26, 27), and GroPIns has been suggested as a possible chemotherapeutic agent (16). Furthermore, GroPIns concentrations vary upon cell transformation even if there is not a clear consistent trend (28). Thus, while Ha-ras transformed fibroblasts, Ret/PTC3 transformed thyroid cells and human papillary carcinoma cells are characterized by high GroPIns levels compared to their normal counterpart, this is not the case in thyroid cells transformed by the src or Ha-ras oncogenes (7, 19, 28).

Despite these studies, so far there is no report on intra- and extracellular differences of GroPIns in human cells. For this reason, and in order to test the new analytical procedure, we sought the inside/outside distribution of GroPIns in tumor and non-tumorigenic cell lines derived from the same tissue. The pairs of the cell lines we took into consideration included the human breast carcinoma MDA-MB-231 and the human mammary epithelial MCF10A cell lines, as well as human prostate cancer PC-3 and the immortalized human prostate epithelial PNT2 cell lines. It is worth noting that MDA-MB-231 cells have been originally used for testing the ability of GroPIns to inhibit *in vitro* extracellular matrix invasion (16). The different cell lines were plated to reach a confluence of about 70–80% after 24 h. At this time, both cells and culture media were collected and the cell number counted. The GroPIns present in the extracellular medium and in the cell pellet was extracted using the SPE method and analyzed in technical triplicate by UPLC-MS/MS as described above. The total amount of GroPIns produced by the cells was calculated by the mathematical sum of intracellular and extracellular values.

**Figure 2:**
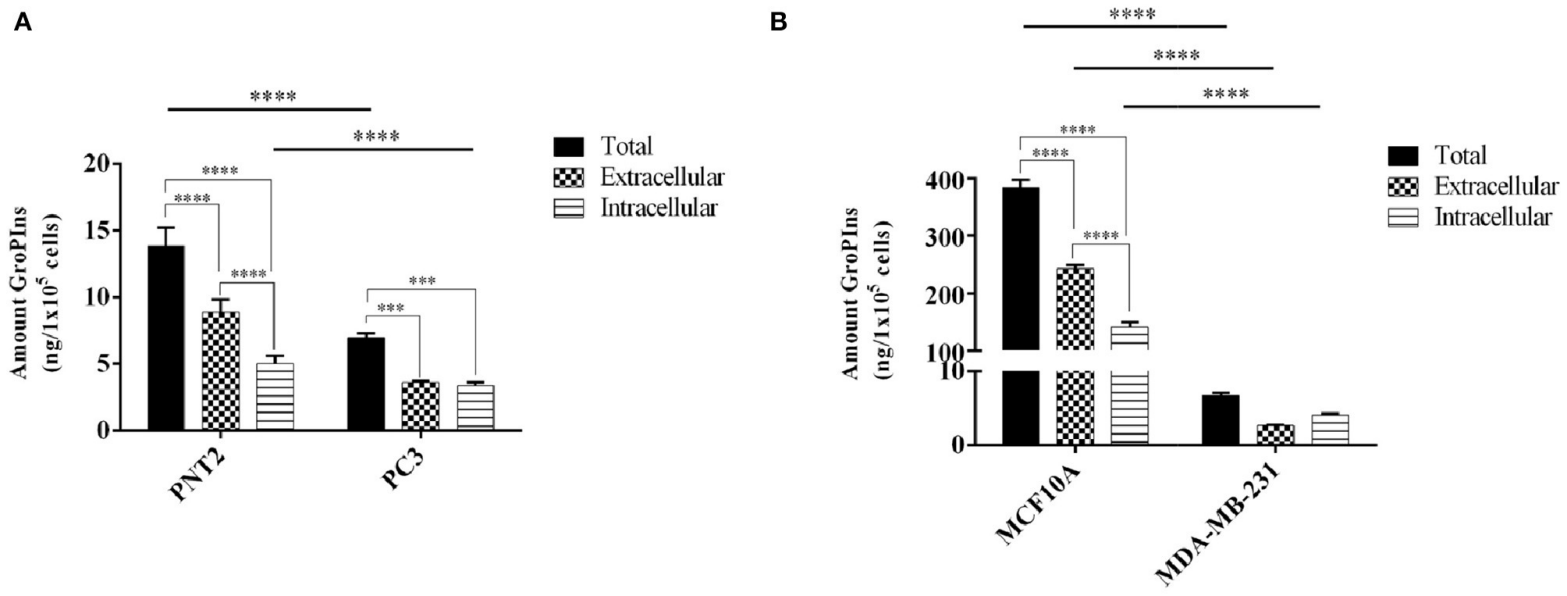
GroPIns levels in non-tumorigenic and tumor-derived cell lines. GroPIns levels on **(A)** human prostate immortalized PNT2 and tumor-derived PC3 prostate cell lines, and **(B)** human mammary epithelial MCF10A and tumor-derived MDA-MB-231 cell lines. Quantitative data were obtained by external calibration method and normalized by 1 × 10^5^ cells. Total levels correspond to the mathematical sum of intracellular and extracellular values. Measurements were made in triplicate (*n* = 3). ****p ≤ 0.0001; ***p ≤ 0.001.

As reported in Figure 2, the immortalized prostate and breast cells PNT2 and MCF10A produced more GroPIns than the respective tumor-derived counterparts PC-3 and MDA-MB-231. In fact, the total amount of GroPIns (mathematical sum of intracellular and extracellular values) produced in PNT2 cells was about double the total concentration present in PC3 cells (Figure 2A), while the difference was even more evident in PNT2 cells that contained on average more than thirty times more GroPIns than PC-3 (Figure 2B). Furthermore, the immortalized prostate and breast cells showed the amount of GroPIns higher in the extracellular medium (63.83% in PTN2 and 63.27% in MCF-10A), while in MDA-MB-231 cells actually displayed an opposite pattern (66.5% intracellular), and PC3 displayed a balanced distribution of this metabolite between the inner and outer compartments (Figures 2A,B).

In absolute terms, the intracellular and extracellular concentrations of GroPIns varied greatly among the different cell lines. In fact, we measured 5.00 ± 0.49 and 140.97 ± 7.24 ng/10^5^ cells for intracellular GroPIns in immortalized lines PNT2 and MCF10A, respectively. With the same cell lines, the extracellular concentration of the analyte was 8.84 ± 0.78 ng/10^5^ cells (61.89 ± 5.49 ng/mL) and 242.80 ± 4.92 ng/10^5^ cells (46.13 ± 0.93 ng/mL). Within the tumor-derived cell lines, we found 3.35 ± 0.20 and 4.05 ± 0.20 ng/10^5^ cells as intracellular pool of PC3 and MDA-MB-231, and 3.62 ± 0.69 and 2.66 ± 0.08 ng/10^5^ cells in the extracellular medium (28.95 ± ng/mL for PC3; 15.45 ± 0.47 ng/mL for MDA-MB-231). In consideration of these results and on the basis of the detection limit of 3 ng/mL, we reasonably concluded that the current method requires at least 5 × 10^5^ cells for a reliable analysis. From a functional point of view, it should be also noted that, while the intracellular GroPIns is referred to the amount of the analyte at a specific time (24 h after plating), the extracellular level is the result of a cumulative process due to the release of proliferating cells in 24 h and includes catabolism and re-uptake of this metabolite.

### Effect of PLA_2_ Inhibition on Extracellular and Intracellular GroPIns

The A375MM cell line derives from a human metastasizing melanoma and has been previously used for functional studies of GroPIns (16). LC-MS/MS analysis also showed a higher concentration of this metabolite in this cell line in comparison with other cell lines (9). This makes A375MM an appropriate benchmark for further testing the new method. As we observed that activation of PLA_2_IVα by ionomycin-dependent influx of Ca^++^ can only slightly increase the level of GroPIns, we also measured the distribution of this metabolite in A375MM after treatment with a cPLA_2_α inhibitor that is expected to reduce the production of GroPIns from phosphatidylinositol (25, 29).

**Figure 3:**
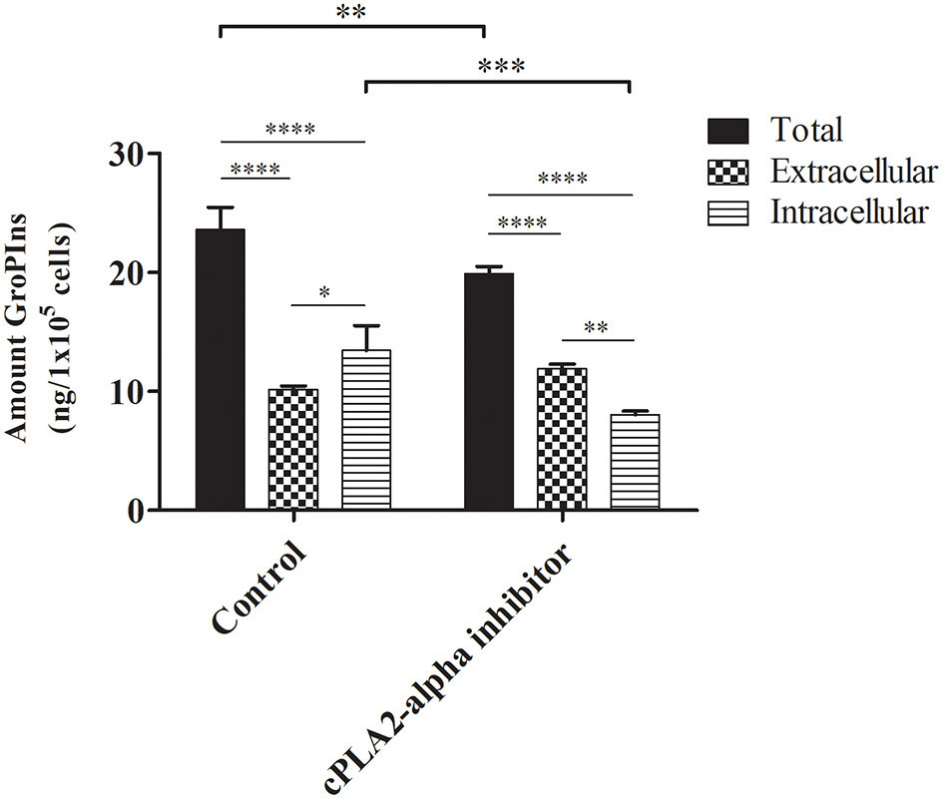
Effect of cPLA_2_α inhibition on GroPIns intracellular and extracellular levels. A375MM cells were either untreated or exposed for 16 h to 0.5 μM cPLA_2_α inhibitor. Quantitative GroPIns data were obtained by external calibration method and normalized per 1 × 10^5^ cells. Total levels correspond to the mathematical sum of intracellular and extracellular values. Measurements were made in triplicate (*n* = 3). ****p ≤ 0.0001; ***p ≤ 0.001; **p ≤ 0.01; *p ≤ 0.05.

In untreated cells, the intracellular amount of GroPIns was 13.43 ± 1.70 ng/10^5^ cells, in agreement with our previous MS measurements (9). In line with the other tumor-derived cell lines reported above, analysis of the extracellular levels (10.15 ± 0.23 ng/10^5^ cells; 26.12 ng/mL) confirmed a preferential intracellular location of this metabolite (13.43 ± 1.70 ng/10^5^ cells) (Figure 3). In the presence of the cPLA_2_α inhibitor (0.5 μM for the last 16 h of culture) we observed the expected decrease of the total amount of GroPIns (mathematical sum of intracellular and extracellular values) from 23.57 ± 1.53 ng/10^5^cells in control cells to 19.88 ± 0.50 ng/10^5^ cells in cells cultured in the presence of cPLA_2_α inhibitor (Figure 3). This response is consistent with the previously observed 20% reduction of GroPIns upon cPLA_**2**_α inhibition in macrophages (29), thus providing a robust evidence of the reliability of the LC-MS/MS method for the quantitation of this metabolite. The effect of cPLA_2_α inhibitor caused a reduction in the intracellular GroPIns levels, due to the block of the enzymatic activity of the cytosolic PLA_2_IVα. This can be interpreted as a hampered synthesis of the compound during the 16 h of treatment. In contrast, the cPLA_2_α inhibitor did not affect the extracellular GroPIns amount, suggesting that the equilibrium due to the GroPIns is not reached during the experimental time (see below).

The Glut2 permease is one of the mammalian carriers responsible for the bidirectional transport of GroPIns (15). This transport follows the chemical gradient until equilibrium is reached. Our results might be explained with a role of Glut2 in non-carcinogenic cells, where the high extracellular levels of GroPIns may depend on active transport of the metabolite through the cell membrane or to the presence of other still not characterized transporters. On the other hand, the significant accumulation of intracellular GroPIns in MDA-MB-231, PC3, and A375MM could involve a decreased transport due to inhibited expression or function of Glut2 that is known to be differently regulated in different cells (30). This hypothesis does not exclude other possibilities related to the regulation of the metabolism of GroPIns. In fact, the increase of the extracellular hydrolysis of GroPIns by the ectoenzymes phosphodiesterases (GDE) 1 and/or 3 can account for these results (31–33). In this view, the tumor cells examined in this study may express a GDE1 or GDE3 more active (or more abundant) than normal cells. Interestingly, the GDE activities could also explain the maintenance of the extracellular GroPIns levels upon cPLA_2_α inhibitor treatment. Similarly, considering the GroPIns synthesis in the cell systems analyzed in this study, a highly expressed (or active) cPLA_2_α could cause the increased total GroPIns levels observed in the non-tumorigenic cells, at variance with previous observations reporting a role of cPLA_2_α activity in transformed cell proliferation (6, 11, 19).

## Conclusions

The development of an improved SPE protocol coupled to UPLC-MS analysis allowed for the first time to accurately quantitate the extracellular levels of GroPIns, revealing an unexpected difference in the tumor and normal cell lines examined in this study. This protocol provides a new tool to address metabolism and effects of GroPIns in different *in vitro* systems and, more important, under *in vivo* diseased states. For example, it is known that the levels of GroPIns upon transformation or differentiation do not follow the same pattern in all cells, but depend on the active signaling pathways including specific oncogenes or cPLA_2_α activities (6, 7, 11, 19, 29). Likewise, it has been shown that GroPIns participates in the resolution of the inflammatory response (17) and that its release from activated macrophages could induce T cell activation via a paracrine mechanism (27, 34). Now it will be possible to mimic these different conditions *in vitro* and investigate the GroPIns mechanism of action by revealing its dynamic behavior and target/s and shedding light on its putative paracrine role.

## Data Availability Statement

The supporting files or minimal data set underlying the findings in our study is within the paper. The MS raw data not contained within the paper are available here: https://cloud.icb.cnr.it/s/ttb5MiZFeimgZ7e. Further inquiries can be directed to Dr. Genoveffa Nuzzo (nuzzo.genoveffa@icb.cnr.it).

## Author Contributions

AMC carried out the experiments and analyzed the data. GN, PI, and AF supervised the study. AV contributed to sample preparation. DB and DC helped supervise the project. GN and AF designed the model. DC encouraged AF to investigate glycerophosphoinositols. DC and AF conceived and planned the experiments. AMC and AF wrote the manuscript. All authors provided critical feedback and helped shape the research, analysis and manuscript.

## Conflict of Interest

The authors declare that the research was conducted in the absence of any commercial or financial relationships that could be construed as a potential conflict of interest.

## Abbreviations

GroPIns: glycerophosphoinositol
GPIs: Glycerophosphoinositols
SPE: Solid Phase Extraction
UPLC: Ultra Performance Liquid Chromatography.
