# Supplementary material for: Direct LC-MS/MS Analysis of Extra- and Intracellular Glycerophosphoinositol in Model Cancer Cell Lines
Source: Front Immunol. 2021 Mar 2;12:646681. doi: 10.3389/fimmu.2021.646681 (PMC7960645; doi:10.3389/fimmu.2021.646681)
Supplement: Supplementary file 1 [file Image_1.pdf]

SUPPLEMENTARY DATA

Direct LC-MS/MS analysis of extra- and intra-cellular glycerophosphoinositol in model cancer cell lines

Ana Margarida Campos[1,2], Genoveffa Nuzzo[1], Alessia Varone[2], Paola Italiani[2], Diana Boraschi[2], Daniela Corda[2,3], Angelo Fontana[1,4*]

[1]Consiglio Nazionale delle Ricerche, Institute of Biomolecular Chemistry, Via Campi Flegrei 34, IT-80078, Pozzuoli, Napoli, Italy.

[2]Consiglio Nazionale delle Ricerche, Institute of Biochemistry and Cell Biology, Via Pietro Castellino 111, IT-80131, Napoli, Italy

[3]Consiglio Nazionale delle Ricerche, Department of Biomedical Sciences, Piazzale Aldo Moro 7, 00185 Rome, Italy

[4] University of Naples Federico II, Department of Biology, Via Cinthia – Bld. 7, 80126 -Napoli, Italy

Figure S1. Replicates of Calibration curves (**A** – **E**) obtained during the development of the SPE elution protocol in the range of 3 – 3000 ng/mL of GroPIns.

A)

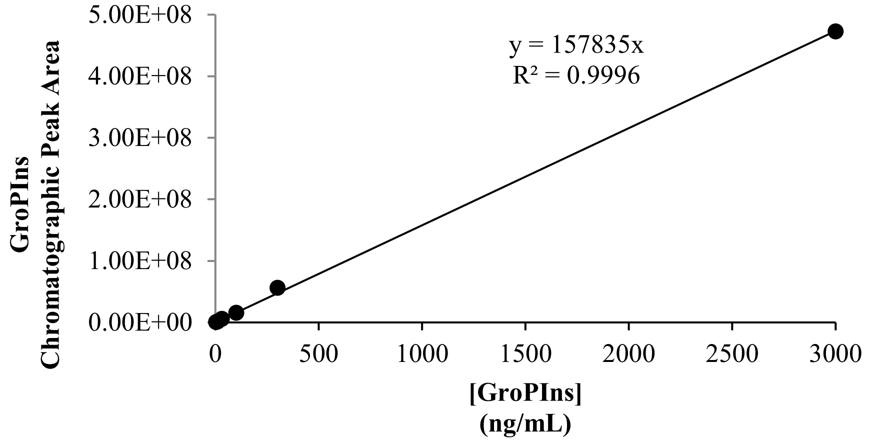

B)

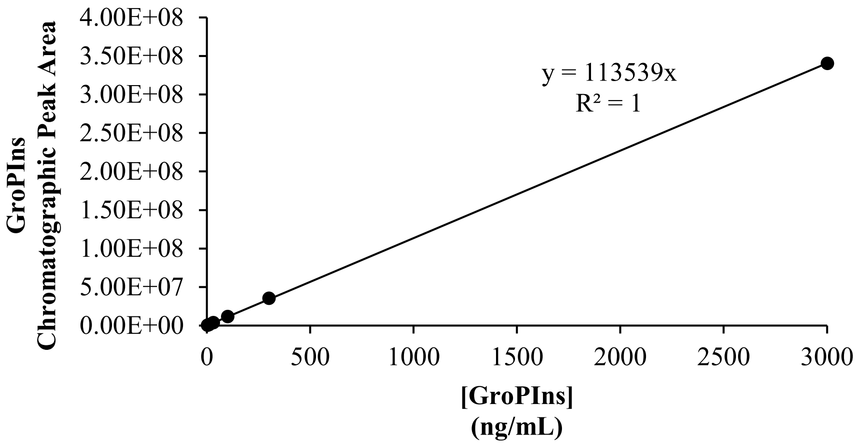

GroPIns
Chromatographic Peak Area
4.00E+08
3.50E+08
3.00E+08
2.50E+08
2.00E+08
1.50E+08
1.00E+08
5.00E+07
0.00E+00
y = 113539x
R² = 1
0
500
1000
1500
2000
2500
3000
[GroPIns]
(ng/mL)

C)

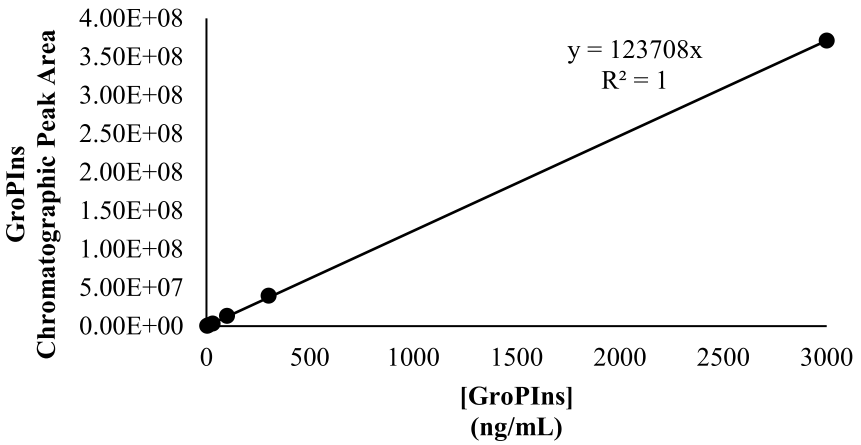

GroPIns
Chromatographic Peak Area
4.00E+08
3.50E+08
3.00E+08
2.50E+08
2.00E+08
1.50E+08
1.00E+08
5.00E+07
0.00E+00
0
500
1000
1500
2000
2500
3000
y = 123708x
R² = 1
[GroPIns]
(ng/mL)

D)

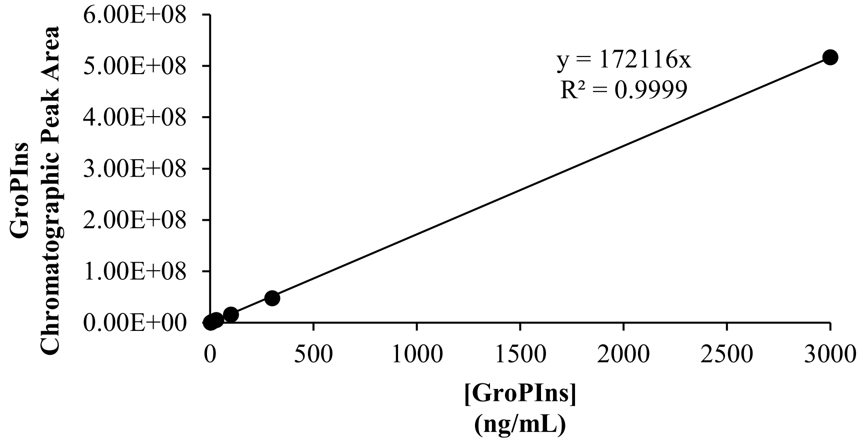

6.00E+08
5.00E+08
4.00E+08
3.00E+08
2.00E+08
1.00E+08
0.00E+00
GroPIns
Chromatographic Peak Area
y = 172116x
R² = 0.9999
0
500
1000
1500
2000
2500
3000
[GroPIns]
(ng/mL)

E)

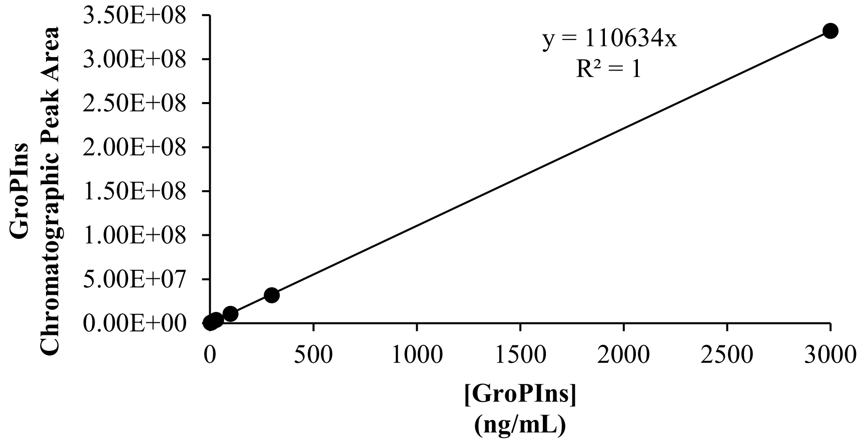
y = 110634x
R² = 1
GroPIns
Chromatographic Peak Area
3.50E+08
3.00E+08
2.50E+08
2.00E+08
1.50E+08
1.00E+08
5.00E+07
0.00E+00
0
500
1000
1500
2000
2500
3000
[GroPIns]
(ng/mL)
